# The Law of the Similars

**Published:** 1881-03

**Authors:** 


### HOMŒOPATHIC PHYSICIAN.

A MONTHLY JOURNAL OF MEDICAL SCIENCE.

"If our school ever gives up the strict inductive method of Hahnemann, we are lost, and deserve to be mentioned only as a caricature, in the history of medicine." — CONSTANTINE HERING.

Vol. I. MARCH, 1881. No. 3.

### EDITORIAL.

THE LAW OF THE SIMILARS. — The title of "homœopathist" should be synonymous with that of a firm, honest belief in the law of nature,—*Similia Similibus Curantur.* But as it is not so, a short discussion of this law will not be inappropriate in this journal, especially as we declare this law to be the chief and central "plank" in our "platform."

It is scarcely credible that one who has seen the law of the similars applied successfully upon hundreds of pathologically differing diseases by means of hundreds of different drugs can harbor a doubt as to its truth; yes, we repeat, such skepticism is scarcely comprehensible. And the only cause for any honest doubts on the part of professed homœopaths must be that the skeptics have not applied the law as correctly as the believers.

Is truth law, or are guess and empiricism to rule? The allopath declares there is no law, because, forsooth, he has not yet found one; no proof is adduced in favor of this opinion. The mongrel homœopath sillily proclaims there is a law for easy simple diseases while only empiricism can cope with the graver forms of disease. In other words, a law is good enough for simple cases, but for real difficult work, guesses and individual opinion are best. The pure homœopath claims there is one all-embracing, never-changing, law governing the administration of drugs. Now, which is correct; no law, a single law, or part law part empiricism? Obviously the question to be decided is between law or no law; the mongrel position of part law and part empiricism being too nonsensical to be entertained for a moment. Then the question to be considered narrows down to the old one long debated by the two opposing schools of medicine. The allopath claiming that a single definite law, governing therapeutics is absurd. The homœopath claiming there is one such law, and shows how it acts in numberless cases. Yet the allopath while denying our law, is fast adopting its logical corollaries.

If nature has laws—each supreme in its sphere—governing all its departments such as physics, astronomy, chemistry, etc., why should it be probable or even *possible*, that there should be none governing the action of drugs? Why should this one sphere of nature's action be left ungoverned by law? Can any one suggest a plausible reason? If drug action is governed by nature—and must it not be ruled by something? man cannot rule it—then the following are to be considered:

1st. There must be one or more laws in this sphere.

2d. If only one, that one must be supreme; that is it must cover the whole ground.

3d. If there could be two, they must be similar or dissimilar.

4th. If two similar, they must be complementary one to the other.

5th. If two dissimilar, they must antagonize one another.

6th. As laws of nature need no assistance—each being supreme in its *sphere*—nor can they antagonize, suppositions four and five cannot exist.

Therefore the only tenable supposition is that of one law. This is the position of all true, honest homœopaths. To them the law of the Similars, enunciated by Hahnemann, is *the* law of therapeutics.

Dr. J. H. Bennett, of England, speaking of medicine (allopathic) lacking a scientific basis, said: "Medicine, then, in its present state, possesses no primitive fact. But is it not possible that it may do so at some future time? During the many ages that existed before Newton, physical science was as inexact as that of physiology now. Before the time of Lavoisier, chemistry, like physiology, consisted of nothing but groups of phenomena. The sciences went on gradually advancing, however, and accumulating facts, until philosophers appeared who united these facts under *one* law*. So medicine, we trust is destined to advance, and one day another Newton, another Lavoisier, may arise whose genius will furnish our science with its primitive fact, and stamp upon it the character of precision and exactitude."

Pure homœopaths claim that the genius of Hahnemann has already furnished medical science with its primitive fact and stamped upon it the character of precision and exactitude. They claim this and nothing more. Is this claim "absurd" or nonsensical; is it "restricting;" does it "abridge one's usefulness;" is it "hero-worship?"

Upon this primitive fact a new materia medica has been built; a materia medica so useful that the allopath slyly adopts its methods: upon this primitive fact a pathology has begun to rear itself and, although yet in its infancy, the "*regular*" (?) disdains not to purloin its teachings: upon this primitive fact a posology has been established which friends and foes utilize. Are not these few straws indicative of a true current? Do they prognosticate truth or falsehood; the real or the visionary?

The allopath may be pardoned for not correctly interpreting

---

* The mongrels will please note this sentence; "united these *facts* under *one law*,"—Ed.

these signs and for not crediting the truth of Hahnemann's discoveries, but no sensible excuse can be framed for the homœopath; he is *supposed* to have read Hahnemann's works, to have experimented as *Hahnemann directed*, and, obtaining practical proof of the truth of his assertions, to have been convinced. Hahnemann said: prove your drugs on the healthy, administer the most similar remedy singly and in the smallest dose that (you think) will cure, leave sufficient space between doses for each to act to its fullest extent; do these things and you will cure your patient. These directions are plain, are easily tried; have those who complain of our law's inefficiency tried this experiment *just as Hahnemann directed?* Has the alternator of drugs tried the single remedy by these simple rules; has the admirer of crude doses tried the minimum dose by these rules? have the advocates of palliatives, of cathartics, etc., *fairly tried these rules and failed?* If so do they charge the failure to fallible man, or to infallible law? Or do they modestly assert there is no such word as fail in *their* lexicons hence *their* failures must be due to weak law!

What are the arguments adduced in favor of the position occupied by the mongrels?

Let us state again their peculiar position: they say the law of the similars is a convenient rule, a rule generally but not always applicable, a rule applying to slight cases but not to grave ones. Such ideas are nonsensical! But, some one may say, the mongrels make no such assertions as these you put into their mouths. We think they do affirm just such ideas by *practice* if not by voice or pen. Do they not recommend quinine for extraordinarily wicked cases of intermittent? do they not advise various and sundry adjuvants for the graver cases of diphtheria; is it not in their dangerous cases that they alternate their drugs; is it not into their "prostrated" patients that they pour their tonics? Who ever heard a mongrel recommend heroic measures for simple, easy cases? Oh! no, there the weak, *fallible* law of nature will suffice, but in grave, serious cases man's *infallible* (!) judgment must be summoned to the rescue! Is it not so?

Have any of these gentlemen who are nominally homœopaths and practically anything but homœopaths, have any of these gentlemen ever adduced any inductive reasoning in favor of their views; have they ever offered any practical demonstration of its superiority over the law of the Similars?

Is not their method rather that of which a noted allopath says: "the old and tried method in therapeutics is that of empiricism, or, if the term sound harsh, of clinical experience.... It is evident this is not a new path, but a highway already worn with the eager but weary feet of the profession for two thousand years"! And it is into this old path that the mongrels would have homœopathy advance (!) that they may leave "antiquated dogmas," cast off "restrictive rules," discard foolish "hero-worship," and wander into the bogs and quagmires of empiricism, where no law limits one's "freedom of medical opinion and action"! Yes, these *reformers* (!) would have homœopathy—young, vigorous, life-giving as she is—turn back, desert her beacon-light of law, and grope in the darkness of this valley of the shadow of death, a valley well worn by blind leaders of the blind, and well strewn by empiricism's victims!

Because this retrograde movement is resisted by pure homœopaths, it is claimed that they desire to rule, to dictate, or to break up the school, etc.

On the other hand, under the guidance of our law, our "primitive fact," pathology, physiology, and therapeutics will in future years attain precision and exactitude. And the facilities for applying our benign law will be elaborated and increased, until we finally attain that almost mathematical precision predicted for our healing art by Hahnemann. This means that we shall be able to save life, to ameliorate suffering, to curtail hereditary disease; all these will be accomplished by the homœopathy of Hahnemann, but *never* by its mongrel caricature.

Which shall we choose: shall we wander back into empiricism, be lost to usefulness, and live as "a caricature, in the history of medicine," or shall we advance through "*the strict inductive method of Hahnemann*" to victory and honor?

All these things—precision and exactitude of pathology, of physiology and of therapeutics; mathematical certainty in the application of our medicines—can be achieved by a strict following out of our law, *if we possess one.* If the law of the similars is not a law of nature *then* we are simply—quacks; our medicines are useless; our theories, absurd; our medical schools, merely eclectic, and the sooner we are exposed the sooner will honesty achieve a necessary vindication. But honesty suffers no wrong from pure homœopathy, for we have a law, a grand beneficent law of nature. That the law of the similars is a law of nature no one can honestly doubt who has faithfully and carefully tried it, as *Hahnemann directed.* Even to those of the laity who cannot investigate this law by practical experiment, there is a mass of *a priori* testimony sufficient to convince any one. The wonderful increase of the profession and the advocacy of our system by the educated of all climes; the most recent investigations of scientists but serve as our unwilling witnesses; the adoption of our medicines, doses and theories by the allopath—all these furnish ample testimony of the truth of our law. Such being our position, let us strive earnestly to perfect this law; let us not sell our birthright for a mess of potage; let us not give our patients a stone when they ask for bread. Nor is this birthright without its responsibilities, for we know that he to whom much is given of him is much expected.

There must ever be strife between truth and error; between law and empiricism. As a celebrated physician has said in another connection; "I trust I have made the issue perfectly distinct and intelligible. And let it be remembered that this is no subject to be smoothed over by nicely adjusted phrases of half assent and half censure, divided between the parties. The balance must be struck boldly and the result declared plainly. . . . Let it be remembered that *persons* are nothing in this matter; there is no quarrel here between *men*, but there is a deadly incompatability and exterminating warfare between doctrines. Let the men who mould opinion look to it; for, if there is any *voluntary* blindness, any *interested* oversight, any *culpable* negligence, in such matters and the facts be publicly known and appreciated, the carriers of woe must look to God for pardon for man will never forgive them."

